# Widespread Pain Phenotypes Impact Treatment Efficacy Results in Randomized Clinical Trials for Interstitial Cystitis/Bladder Pain Syndrome: A MAPP Network Study

**DOI:** 10.21203/rs.3.rs-2441086/v1

**Published:** 2023-02-23

**Authors:** John Farrar, Kenneth Locke, J Clemens, James Griffith, Steven Harte, Ziya Kirkali, Karl Kreder, John Krieger, H. Henry Lai, Robert Moldwin, Chris Mullins, Bruce Naliboff, Michel Pontari, Larissa Rodríguez, Anthony Schaeffer, Alisa Stephens-Shields, Siobhan Sutcliffe, Bayley Taple, David Williams, J Landis

**Affiliations:** University of Pennsylvania Perelman School of Medicine; University of Pennsylvania, Perelman School of Medicine; University of Michigan Medical School; Northwestern University; University of Michigan; National Institute of Diabetes and Digestive and Kidney Diseases, National Institutes of Health; University of Iowa Carver College of Medicine; University of Washington School of Medicine; Washington University School of Medicine; Hofstra North Shore-LIJ School of Medicine; National Institute of Diabetes and Digestive and Kidney Diseases, National Institutes of Health; UCLA; Temple University Lewis Katz School of Medicine; NewYork-Presbyterian/Weill Cornell Medical Center; Northwestern University; University of Pennsylvania; Washington University School of Medicine, St. Louis; Northwestern University Feinberg School of Medicine; University of Michigan Medical School; University of Pennsylvania Perelman School of Medicine

## Abstract

Clinical trials of pain are notoriously difficult and inefficient in demonstrating efficacy even for known efficacious treatments. Determining the appropriate pain phenotype to study can be problematic. Recent work has identified the extend of widespread pain as an important factor in the likelihood of response to therapy, but has not been tested in clinical trials. Using data from three previously published negative studies of the treatment of interstitial cystitis/ bladder pain with data on the extent of widespread pain, we examined the response of patients to different therapies base on the amount of pain beyond the pelvis. Participants with predominately local but not widespread pain responded to therapy targeting local symptoms. Participants with widespread and local pain responded to therapy targeting widespread pain. Differentiating patients with and without widespread pain phenotypes may be a key feature of designing future pain clinical trials to demonstrate treatments that are effective versus not.

## Introduction

Pain is a subjective experience which is known to have multiple mechanisms for the generation and maintenance of painful syndromes. The resulting heterogeneity of patient populations enrolled in clinical trials is thought to one of the primary issues in conducting clinical trials to demonstrate efficacy even for known efficacious therapies^1,2^. Recent work has identified the extend of widespread pain as an important factor in the likelihood of response to therapy in chronic pelvic pain syndromes including interstitial cystitis/bladder pain syndrome (IC/BPS), but has not been tested in clinical trials.

Interstitial cystitis/bladder pain syndrome (IC/BPS) is estimated to affect 2.9–4.2% of the US population with a high degree of patient morbidity and few effective treatments.^3^ In one of the largest comprehensive cohort studies of patients with pain, the Multidisciplinary Approach to the Study of Chronic Pelvic Pain (MAPP) Research Network has measured and evaluated the importance of a number of factors in the course of the disease over time, including the extent of widespread pain beyond the pelvis,^4–9^ and its association with Chronic Overlapping Pain Conditions (COPCs). COPCs are known to occur in 39% of IC/BPS patients, indicating the degree of pain manifested in other parts of the body.^10,11^ In MAPP patients, the extent of COPCs and pain beyond the pelvic sites reported on a pain body map were found to be contributing factors to IC/BPS symptom severity and lower quality of life.^7^ The MAPP Network has also demonstrated fundamental differences in pain-related brain connectivity (functional MRI scans)^8^, experimental pain sensitivity (quantitative sensory testing)^12^, and immunological factors between patients with localized versus more widespread pain.^13^

Until the end of the 20th century, idiopathic widespread pain was often used as a marker for a “psychogenic” cause of pain due to the limited understanding of the interaction between the central and peripheral nervous system. With the recent rapid growth in knowledge about the complexities of the nervous system and in large part due to the development of functional brain imaging^14^, it is now understood that widespread pain is often a marker of peripheral and central pain pathway dysfunction deserving of intense basic and clinical research to promote improved care for patients with chronic pain.^15^ A growing number of clinical trials have explored potential therapies for widespread pain,^16,17^ but almost none have examined its impact on the outcome of trials for specific pain syndromes and other symptoms.

This has come about in a setting where advances in personalized therapeutics require increasingly more precise characterization of individual phenotypes, especially in similar pain syndromes with different mechanistic phenotypes.^18,19^ Without adequate phenotyping, testing of mechanistically based therapies can lead to false negative clinical trial results, due to dilution of the potentially responsive phenotype. Precise characterization of individual phenotypes will have an important impact on future clinical trial designs meant to identify effective targeted therapies for disorders that frequently include patients with diverse symptoms.^20,21^ To extend the MAPP cohort study findings to clinical trial data, we used the availability of individual patient data from previously conducted randomized clinical trials (RCTs) of treatments for IC/BPS to explore the impact of the extent of widespread pain on treatment responses in RCTs.

## Results

### Demographics

Participant characteristics and baseline measures,^22–24^ are summarized by pain widespreadness subgroups in Table 1 using the package tableone in R.^34^ Average age ranged from 37.3 to 49.9 years, female sex from 73% to 96%, and Caucasian race from 60% to 89% across subgroups and RCTs. Based on these differences, analyses were adjusted for age, sex, and race (Table 1).

### Treatment Response in Combined Sample

Most treatment effects for absolute change in NRS outcomes in the full study population were not significant (α=0.05), with the exception of urgency (change in urgency=−0.57, 95% CI [−1.09,−0.04], p=.034) in the BCG trial and frequency in the amitriptyline trial (change in frequency=−0.78, 95% CI [−1.38,−0.18], p=.011) (Extended Table 1). The treatment effects for the GRA endpoint were statistically significant for all three trials: PPS/Hydroxyzine (log odds ratio=1.09, 95% CI [0.12,2.06], p=0.027), BCG (log odds ratio=0.67, 95% CI [0.01,1.33], p=0.048) and amitriptyline (log odds ratio=0.67, 95% CI [0.15,1.19], p=0.012), when analyzed using multiple imputation model approaches to handle missing data. This contrasts with the more conservative approach, used in the respective publications, of assuming patients missing GRA data at study endpoint are non-responders: PPS vs. No PPS (34% response vs. 18%, p=0.064), Hydroxyzine vs. No Hydroxyzine (31% response vs 20%, p=.260), BCG (21% response for BCG vs. 12% for placebo, p=0.062), and amitriptyline (55% response for amitriptyline vs. 45% for control, p=0.12).

### Treatment Response: Stratified by Pain Widespreadness Subgroups

#### Pentosan Polysulfate Sodium (PPS)/ Hydroxyzine study

**Figure 1:**
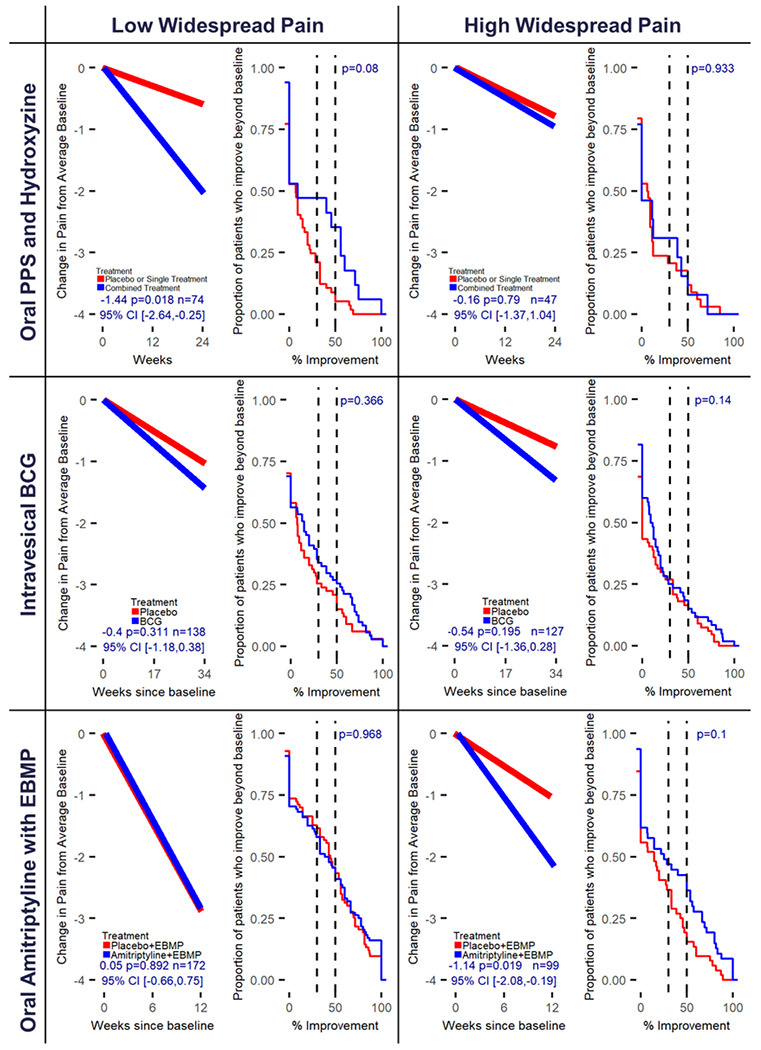
Predicted change and observed percentage improvement in pelvic pain from baseline by RCT and pain widespreadness. The mean trajectory plots (left within cell) represent the estimated change in pelvic pain from average baseline for the average participant within RCT accounting for baseline outcome, age, race, and sex. Treatment effect, corresponding p-value and 95% confidence interval are displayed within plot. Mean trajectories were derived from the primary analysis models for change in outcome at end of study from average baseline utilizing multiple imputation for missing data. The 1 -ECDF plots (right within cell) represent the proportion of patients whose observed percentage improvement in end of study outcome from average baseline is beyond the value on x-axis. Patients missing end of study data have an observed percentage change of 0. P-value for testing the difference in observed percentage improvement curves for treatment (Blue) and control (Red) are displayed within plot and are derived from the non-parametric Wilcoxon Rank-Sum test (see Extended Methods).

**Figure 2:**
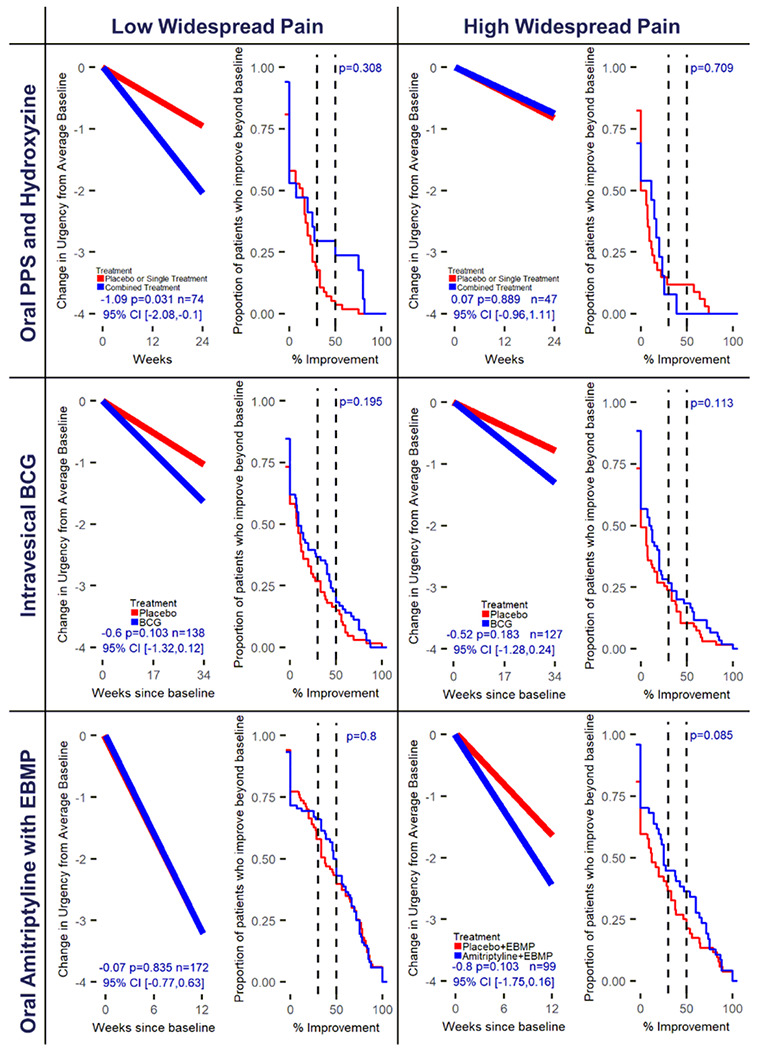
Predicted change and observed percentage improvement in urinary urgency from baseline by RCT and pain widespreadness. The mean trajectory plots (left within cell) represent the estimated change in urinary urgency from average baseline for the average participant within RCT accounting for baseline outcome, age, race, and sex. Treatment effect, corresponding p-value and 95% confidence interval are displayed within plot. Mean trajectories were derived from the primary analysis models for change in outcome at end of study from average baseline utilizing multiple imputation for missing data. The 1-ECDF plots (right within cell) represent the proportion of patients whose observed percentage improvement in end of study outcome from average baseline is beyond the value on x-axis. Patients missing end of study data have an observed percentage change of 0. P-value for testing the difference in observed percentage improvement curves for treatment (Blue) and control (Red) are displayed within plot and are derived from the non-parametric Wilcoxon Rank-Sum test (see Extended Methods).

Neither of the single treatments, PPS or hydroxyzine, were statistically different from placebo for either pelvic pain or urinary urgency outcomes in both pain widespreadness subgroups. Therefore, the single treatment and placebo arms were combined to form a comparator arm to the combination PPS/Hydroxyzine therapy. The combination treatment versus the comparator arm was statistically significant for pelvic pain (change in pelvic pain=−1.44, 95% CI [−2.64,−0.25], p=0.018) (Figure 1, Table 2), and urinary urgency (change in urinary urgency=−1.09, 95% CI [−2.08,−0.10], p=0.031) (Figure 2, Table 2), but only for patients with low pain widespreadness. In the 1 –ECDF curves for observed change in pelvic pain (Extended Figure 8), the number of participants achieving 50% improvement or greater for the combined therapy compared to the control arm were 35% vs 9% in the low pain widespreadness subgroup, with nearly overlapping response rates in the high pain widespreadness subgroup.

#### Intravesical Bacillus Calmette-Guerin (BCG) study

Treatment response was not statistically significant for change in pain or urgency in either subgroup (Figures 1b and 2b) but was for GRA response (Table 2) comparing BCG treatment to placebo only in the high widespread pain group. All 1 – ECDF curves overlapped indicating no difference in observed improvement (Figures 1–2).

#### Amitriptyline:

**Figure 3:**
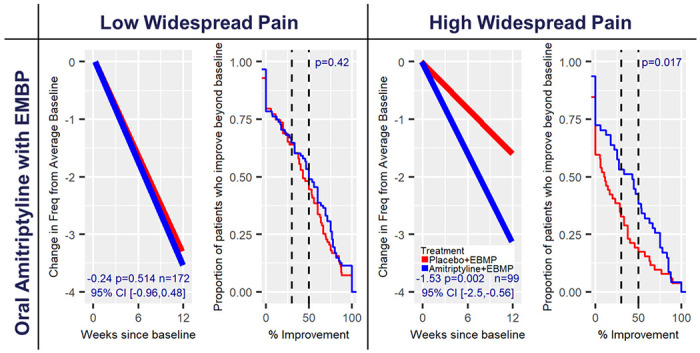
Predicted change and observed percentage improvement in urinary frequency from baseline for amitriptyline trial by pain widespreadness. The mean trajectory plots (left within cell) represent the estimated change in urinary frequency from average baseline for the average participant within the amitriptyline train accounting for baseline outcome, age, race, and sex. Treatment effect, corresponding p-value and 95% confidence interval are displayed within plot. Mean trajectories were derived from the primary analysis models for change in outcome at end of study from average baseline utilizing multiple imputation for missing data. The 1-ECDF plots (right within cell) represent the proportion of patients whose observed percentage improvement in end of study outcome from average baseline is beyond the value on x-axis. Patients missing end of study data have an observed percentage change of 0. P-value for testing the difference in observed percentage improvement curves for treatment (Blue) and control (Red) are displayed within plot and are derived from the non-parametric Wilcoxon Rank-Sum test (see Extended Methods)

Amitriptyline compared to placebo, with all patients receiving an IC/BPS focused EBMP, demonstrated a statistically significant difference for change in pelvic pain (change in pelvic pain=−1.14, 95% CI [−2.08,−0.19], p=0.019) (Figure 1), urinary frequency (change in urinary frequency=−1.53, 95% CI [−2.50,−0.56], p=0.002) (Figure 3) and the GRA response (log odds ratio=1.18, 95% CI [0.29,2.07], p=0.009) outcomes for patients with high widespread pain (Table 2). Amitriptyline with EBMP response was similar in both widespread pain subgroups; however, placebo with EBMP was similarly effective in the low widespread pain group leading to non-significant treatment differences (Figure 3) but much less effective in the high widespread pain group leading to the statistically significant differences. Comparing observed percentage change in the 1 – ECDF curves between subgroups, differences between treatments were observed for the high pain widespreadness subgroup for both pelvic pain and frequency outcomes. For frequency, the difference in proportion of patients with 50% improvement or higher within the high widespreadness subgroup was 43% vs 19% for amitriptyline with EBMP compared to placebo with EBMP (Extended Figure 10).

## Discussion

The primary finding from our re-analysis approach is the influence of the widespread pain phenotype on treatment efficacy for patients with IC/BPS. We identified symptom improvement from the PPS/hydroxyzine combination only in patients with low widespread pain. Amitriptyline demonstrated similar treatment improvement responses in both widespread pain groups, but only statistically significant in the high widespread pain group because the IC/BPS focused EBMP control group demonstrated similar efficacy in the low widespread group, but much lower benefit in the high widespread group. To the best of our knowledge, this study is the first to support the importance of widespread pain on treatment efficacy for patients with IC/BPS and supports the likelihood of a similar impact on other centrally vs peripherally pain syndromes.

Importantly, the effect of widespread pain on response to treatment was different in the three studies, but consistent with the putative mechanism of action of the treatment. PPS/hydroxyzine is thought to target localized pelvic symptoms specifically. The combination therapy was only superior in the absence of widespread pain, suggesting that the local benefit was not adequate in patients with pain extending beyond the pelvis. In contrast, the noradrenergic/serotonergic effects of amitriptyline have been demonstrated to provide benefit in generalized pain^35,36^, and the anti-cholinergic effects are thought to reduce some urinary symptoms.^37^ Amitriptyline demonstrated an effect in both the high and low widespread pain phenotypes; however, the EBMP behavioral therapy alone was effective for both pain, urinary, and frequency symptoms only in the absence of widespread pain. The level of response in the EBMP behavioral control group limits the detection of a statistically significant effect for amitriptyline in the low widespread group, despite the same level of response in all patients. BCG instillation is a local therapy with a low level of benefit for both the low and high widespread pain groups for pain or urgency. Without consideration of the widespread pain phenotype, the negative results of the analysis of the whole population may not identify potentially effective therapies for a group of patients for whom there are few treatments.

The heterogeneity of treatment effect is known to be important in evaluating clinical trials. A baseline risk-based approach defining outcome heterogeneity on identifiable phenotypic characteristics may provide useful insights for the interpretation of previously conducted RCTs and have implications in the design of future clinical studies.^38^ Such a re-analyses approach to defining phenotypes in patients with diabetes has led to better understanding of response in drug vs behavioral therapy.^39^ Specific phenotype-based analyses should be planned a priori to increase the assay sensitivity of the clinical trial.^40^ In addition, our results suggest that the spatial distribution of a patient’s pain should be measured and considered in the choice of therapies used for patient care as well.^40^

We recognize several important limitations of our study. Although our results support the importance of overall pain widespreadness in treatment response these findings need to be confirmed in future clinical trials and other prospective studies as subgroup analyses have well-known limitations.^38,41^ It will be important to conduct future studies using sensory testing methods, in addition to patient reports of pain, to differentiate underlying processes that may maintain widespread pain, because none of our earlier studies included such measures. The differences between our three studies involve more than just the focus of the therapies. The amitriptyline study enrolled new IC/BPS patients; whereas the PPS/hydroxyzine and BCG studies enrolled patients with more chronic conditions who had undergone previous treatment. These differences are also a strength since the presence of widespread pain had a substantial impact on two of the three trials. Lastly, our measure of widespread pain phenotype was limited to five available questions about non-pelvic pain, which may be less robust than a body map. Nevertheless, we detected a substantial difference in treatment response in two of the three studies. A strength of our study is the quality of the trial data, which were collected as part of well-designed, and carefully conducted clinical trials, with rigorous data quality procedures, and limited missing data.

## Conclusions

The present study strongly supports the importance of the baseline identification of patient widespread pain phenotypes that substantially affect the outcomes in two of three previously published RCTs for IC/BPS. The data suggests that treatments focused on urinary and pelvic pain symptoms are more likely to demonstrate benefit in patients where local symptoms predominate. In contrast, patients with widespread pain may require centrally-directed treatments intended for more generalized pain. Our findings support the importance of additional research to identify methods of measuring characteristics that define important symptom phenotypes, including those measures in the design of clinical trials, and making evidence-based decisions about which patients to include in studies of symptomatic therapy, including those with IC/BPS.

## Methods

This study used previously collected and anonymized data and was classified as exempt by our IRB.

### Population: IC/BPS RCTs

Three of the published IC/BPS RCT studies (pentosan polysulfate sodium (PPS)/hydroxyzine^22^, intravesical bacillus calmette-guerin (BCG)^23^, and amitriptyline^24^) collected WSS data, permitting re-analyses incorporating baseline stratification by widespread pain. Patients were >18 years of age, and for the PPS/hydroxyzine and BCG studies, had symptoms for 24 weeks, a pain/discomfort score of >4/9, urinary frequency >11 times in 24-hours for four weeks, and IC/BPS diagnosis verified through cystoscopy and hydrodistension. Patients were followed for 24 weeks for the PPS/hydroxyzine trial and 34 weeks for the BCG trial. The amitriptyline trial focused on untreated IC/BPS patients with at least 6 weeks of symptoms and pain severity and urinary frequency scores of ≥3/10 for at least four weeks. The study did not require a previous IC/BPS diagnosis for inclusion. Amitriptyline doses were titrated from 25 to 75mg, as tolerated, and followed for 12 weeks. All patients in this trial also received a standardized IC/BPS focused education and behavioral modification program (EBMP), including instruction on fluid and food, bladder, and stress management.

### Baseline Stratification by Pain Widespreadness

To construct subgroups with maximal separation of baseline symptoms, an unsupervised consensus clustering (CC) algorithm (ConsensusClusterPlus) in R (version 3.4.1)^26^ was applied over the combined RCT dataset consisting of 16 WSS pain, urinary and frequency symptoms questions and 5 measures of pain beyond the pelvis (headache, backache, chest pain, joints aches, abdominal cramps), each reported on a 0-6 scale from “not-at-all” to “a lot”.^25^ An ideal classification rule using only the 5 WSS measures of pain beyond the pelvis was developed to predict membership to the high widespread pain cluster through logistic regression models with receiver operator characteristics curves (ROC) applied to a harmonized analysis dataset of all three RCT studies.^27,28^ In particular, participants had to have a score of ≥2/6 reported for ≥3 of 5 WSS pain questions to be classified as high widespreadness of pain in our analyses. Further details about consensus clustering and development of widespreadness classification can be found in the Extended Methods and Extended Figures 1–4.

### Outcomes

IC/BPS symptoms of pelvic pain and urinary urgency were measured on a numeric rating scale (NRS), and absolute change was estimated as the difference between the study endpoint measure and the average of the screening and baseline visit measure. Percent change was calculated as the outcome at end of study minus the average of baseline and screening, divided by the outcome and then multiplied by 100 for each NRS scale. A 0-9 NRS scale was used for pain and urinary urgency in the PPS/hydroxyzine and BCG trials; whereas the 0-10 scale was used in the amitriptyline trial which measured urinary frequency in addition. As an alternative to evaluating change in 3 separate IC/BPS symptoms, the original RCT analyses used a global response assessment (GRA) for the primary outcome measure. The 7-point GRA scale, collected at study endpoint, ranged from 1:markedly worse, 2:moderately worse, 3:slightly worse, 4:no change, 5:slightly improved, 6:moderately improved, or 7:markedly improved, with a responder defined as ≥6.

### Analyses

All statistical analyses were implemented within SAS 9.4. The primary modeling was conducted with the three NRS outcomes specified above. All statistical hypothesis tests are two-sided with a level of significance of p=0.05 and were not corrected for multiple comparisons in our analyses. As this work is a reanalysis of clinical trial data, all analyses are post-hoc and results are interpreted as exploratory and hypothesis-generating.

### Absolute Change Modeling for NRS Measures

The primary re-analysis of data from each RCT was designed to detect differential response between treatment arms within widespread pain strata for each NRS measure (pelvic pain, urinary urgency, urinary frequency). The absolute change between the outcome at the end of study and baseline average of outcome at screening and randomization visit was modeled within a GLM, with separate treatment effects for each widespread pain subgroup, and covariate effects for average baseline outcome, age, race, and sex. Negative treatment effects were indicative of symptom improvements. All primary analyses implemented multiple imputation with predictive mean matching and m=100 imputations^29,30^ to impute missing outcome and baseline covariates assuming data are missing at random. Rubin’s rules were used to calculate the final model coefficients and corresponding standard errors.^29–31^ (Extended Methods) Treatment effect heterogeneity between widespread pain subgroups was formally tested within GLM for each NRS measure. Complete case analyses and modeling treatment effects without regard to widespreadness were run as sensitivity analyses (Extended Tables 1–2). Estimated change in NRS measures of treatment and control for each widespreadness subgroup were calculated (Extended Table 3) and displayed along with the mean trajectory plots (Figures 1–3, Extended Figures 5–7) with further details contained in the Extended Methods.

### Global Response Assessment (GRA) Modeling

The second re-analysis modeled responders (GRA=6,7) with logistic regression to detect differential responder rates between treatment arms within widespread pain strata, adjusted for age, race, and sex. Treatment effect heterogeneity in GRA responder rates between widespread pain subgroups was formally tested within the overall GLM.

### Percentage Change Modeling for NRS Measures

Empirical cumulative distribution functions (ECDFs) of observed percentage change from baseline to the end of study for the 3 NRS symptom outcomes were generated by treatment arm within widespread pain subgroups. Patients with missing observations for the end of study outcome were assigned a percentage change of zero. Plots for the inverse (1 – ECDF) display the proportion of patients with a percentage improvement above the value indicated on the x-axis (generated in R with the package ggplot2).^32^ (Figures 1–3, Extended Figures 8–10) A non-parametric Wilcoxon Rank Sum test was implemented to test differences between treatments within widespread pain subgroups given distribution of observed percentage change.^33^ Further details regarding the figures can be found in the Extended Methods.

## Supplementary Material

1

## Figures and Tables

**Table 1.** Summary of demographics and baseline symptoms for the IC/BPS RCTs stratified by low and high pain widespreadness subgroups.

	Oral PPS and Hydroxyzine			Intravesical BCG			Oral Amitriptyline with EBMP		
	Low Pain Widespread	High Pain Widespread	Combined Total	Low Pain Widespread	High Pain Widespread	Combined Total	Low Pain Widespread	High Pain Widespread	Combined Total
**Baseline Data**									
**Demographics**									
Number of Patients: No.	74	47	121	138	127	265	172	99	271
Female: No. (%)	63 (85)	45 (96)	108 (89)	101 (73)	116 (91)	217 (82)	132 (77)	93 (94)	225 (83)
Caucasian: No. (%)	60 (81)	42 (89)	102 (84)	120 (87)	110 (87)	230 (87)	135 (79)	59 (60)	194 (72)
Age: mean (SD)	47.8 (15.0)	43.0 (14.5)	45.9 (14.9)	49.9 (14.4)	45.2 (12.0)	47.7 (13.5)	40.0 (14.6)	37.3 (12.5)	39.0 (13.9)
**Baseline Symptoms: mean (SD)**									
Pain NRS^ab^	5.9 (1.2)	6.2 (1.2)	6.1 (1.2)	6.6 (1.3)	7.0 (1.2)	6.8 (1.2)	5.6 (1.7)	6.3 (1.6)	5.9 (1.7)
Urgency NRS^ab^	6.6 (1.4)	6.5 (1.4)	6.6 (1.4)	6.8 (1.3)	7.2 (1.2)	7.0 (1.3)	6.1 (1.8)	6.8 (1.8)	6.4 (1.8)
Frequency NRS^ab^	NA	NA	NA	NA	NA	NA	6.6 (1.7)	7.2 (1.8)	6.8 (1.7)
IC Problem Index (0-16)	12.3 (3.0)	13.1 (1.9)	12.6 (2.6)	12.4 (2.6)	13.2 (2.1)	12.7 (2.4)	10.7 (2.7)	11.9 (2.9)	11.2 (2.9)
IC Symptom Index (0-20)	13.8 (3.8)	14.5 (2.9)	14.1 (3.5)	14.1 (3.4)	14.8 (3.1)	14.4 (3.2)	11.7 (3.1)	12.9 (3.5)	12.1 (3.3)
Wisconsin IC Score (0-42)	29.4 (8.3)	33.8 (5.2)	31.0 (7.6)	30.2 (7.8)	33.6 (6.5)	31.8 (7.4)	24.3 (8.3)	29.7 (7.6)	26.3 (8.4)
Years of Urinary Symptoms	12.2 (10.1)	11.4 (8.9)	11.9 (9.6)	12.2 (10.7)	15.4 (12.5)	13.7 (11.7)	6.7 (9.5)	7.3 (9.7)	6.9 (9.5)
Years Since IC Diagnosis^c^	5.1 (6.0)	4.8 (4.9)	5.0 (5.6)	5.9 (5.4)	6.5 (6.8)	6.2 (6.1)	1.9 (3.0)	1.8 (4.4)	1.8 (3.6)
Wisconsin Widespreadness Score (0-30)	5.2 (3.6)	14.1 (4.2)	8.7 (5.8)	4.7 (3.1)	14.8 (5.1)	9.6 (6.6)	4.5 (3.1)	14.0 (4.8)	8.0 (6.0)
**Missing Outcomes**									
Missing Data Counts: No. (%)	16 (22)	8 (17)	24 (20)	7 (5)	12 (9)	19 (7)	23 (13)	18 (18)	41 (15)

a Average of the two baseline visits

b (0-9) NRS Scale for Oral PPS/Hydroxyzine and Intravesical BCG Trials. (0-10) NRS Scale for Oral Amitriptyline with EBMP Trial

c Not all patients in Oral Amitriptyline with EBMP Trial were diagnosed with IC

**Table 2.** Observed change in outcome and estimated treatment effects by pain widespreadness subgroup across the IC/BPS RCTs.

	Low Widespreadness			High Widespreadness			
	Complete Cases		Intent to Treat	Complete Cases		Intent to Treat	
	Observed Change: Control mean (SD)	Observed Change: Treatment mean (SD)	Multiple Imputation Model: Treatment^c^	Observed Change: Control mean (SD)	Observed Change: Treatment mean (SD)	Multiple Imputation Model: Treatment^c^	Multiple Imputation Model: Treatment Interaction^d^
**Oral PPS and Hydroxyzine**							
**N**	47	11	74^e^	27	12	47^e^	
Logistic Regression on Log Odds Scale for Binary Outcome							
**GRA Responder** ^a^	11 (23%)	7 (64%)	1.28 (0.059) [−0.05,2.61]^f^	8 (30%)	5 (42%)	0.64 (0.379) [−0.79,2.07]^f^	−0.64 (0.522) [−2.60,1.32]
Absolute Change on NRS Scale							
**Change in Pain NRS** ^b^	−0.68 (1.77)	−2.36 (1.87)	−1.44 (0.018) [−2.64,−0.25]	−0.91 (1.84)	−0.96 (1.84)	−0.16 (0.790) [−1.37,1.04]	1.28 (0.141) [−0.42,2.99]
**Change in Urgency NRS** ^b^	−1.05 (1.48)	−2.05 (1.74)	−1.09 (0.031) [−2.08,−0.10]	−0.80 (1.83)	−0.58 (1.00)	0.07 (0.889) [−0.96,1.11]	1.16 (0.108) [−0.26,2.58]
**Intravesical BCG**							
**N**	64	67	138^e^	62	53	127^e^	
Logistic Regression on Log Odds Scale for Binary Outcome							
**GRA Responder** ^a^	11 (17%)	14 (21%)	0.31 (0.471) [−0.54,1.16]^f^	5 (8%)	13 (25%)	1.18 (0.037) [0.07,2.28]^f^	0.86 (0.225) [−0.53,2.26]
Absolute Change on NRS Scale							
**Change in Pain NRS** ^b^	−00.96 (2.27)	−1.40 (2.61)	−0.40 (0.311) [−1.18,0.38]	−0.86 (1.97)	−1.50 (2.21)	−0.54 (0.195) [−1.36,0.28]	−0.14 (0.807) [−1.27,0.99]
**Change in Urgency NRS** ^b^	−0.98 (2.03)	−1.57 (2.37)	−0.60 (0.103) [−1.32,0.12]	−0.89 (1.96)	−1.48 (2.29)	−0.52 (0.183) [−1.28,0.24]	0.08 (0.880) [−0.97,1.13]
**Oral Amitriptyline with EBMP**							
**N**	75	74	171^e^	44	37	99^e^	
Logistic Regression on Log Odds Scale for Binary Outcome							
**GRA Responder** ^a^	44 (59%)	49 (66%)	0.30 (0.379) [−0.36,0.96]^f^	17 (39%)	25 (68%)	1.18 (0.009) [0.29,2.07]^f^	0.88 (0.118) [−0.22,1.99]
Absolute Change on NRS Scale							
**Change in Pain NRS** ^b^	−2.64 (2.37)	−2.70 (2.43)	0.05 (0.892) [−0.66,0.75]	−1.59 (2.38)	−2.45 (2.62)	−1.14 (0.019) [−2.08,−0.19]	−1.19 (0.049) [−2.37,0.00]
**Change in Urgency NRS** ^b^	−2.81 (2.44)	−3.10 (2.49)	−0.07 (0.835) [−0.77,0.63]	−1.99 (2.72)	−2.96 (2.53)	−0.80 (0.103) [−1.75,0.16]	−0.72 (0.230) [−1.90,0.46]
**Change in Frequency NRS** ^b^	−3.06 (2.35)	−3.52 (2.21)	−0.24 (0.514) [−0.96,0.48]	−1.85 (2.61)	−3.50 (2.44)	−1.53 (0.002) [−2.50,−0.56]	−1.29 (0.036) [−2.49,−0.08]

a No. (%)

b Change is calculated as the outcome at the end of study subtracted from averaged baseline

c Estimated treatment effect (Change in outcome on treatment compared to control within widespreadness subgroup) from multiple imputation model results: Effect (p-value) [95% CI]

d Test for interaction between treatment effect and widespreadness category. Based off of multiple imputation model results. Difference=treatment high widespreadness-treatment low widespreadness (p-value) [95% CI]

e Sample size of treatment and control within widespreadness subgroup under ITT

f Treatment effect log odds ratio from multiple imputation model results: Log odds ratio (p-value) [95% CI]
