# Supplementary material for: Widespread Pain Phenotypes Impact Treatment Efficacy Results in Randomized Clinical Trials for Interstitial Cystitis/Bladder Pain Syndrome: A MAPP Network Study
Source: Res Sq. 2023 Feb 23:rs.3.rs-2441086. Preprint. [Version 1] doi: 10.21203/rs.3.rs-2441086/v1 (PMC9980200; doi:10.21203/rs.3.rs-2441086/v1)
Supplement: 1 [file NIHPPrs2441086v1-supplement-1.pdf]

Extended Data Figures

Extended Figure1. Unsupervised consensus clustering algorithm applied to a combined IC/BPS baseline dataset of N=649 patients across 21 scaled symptoms

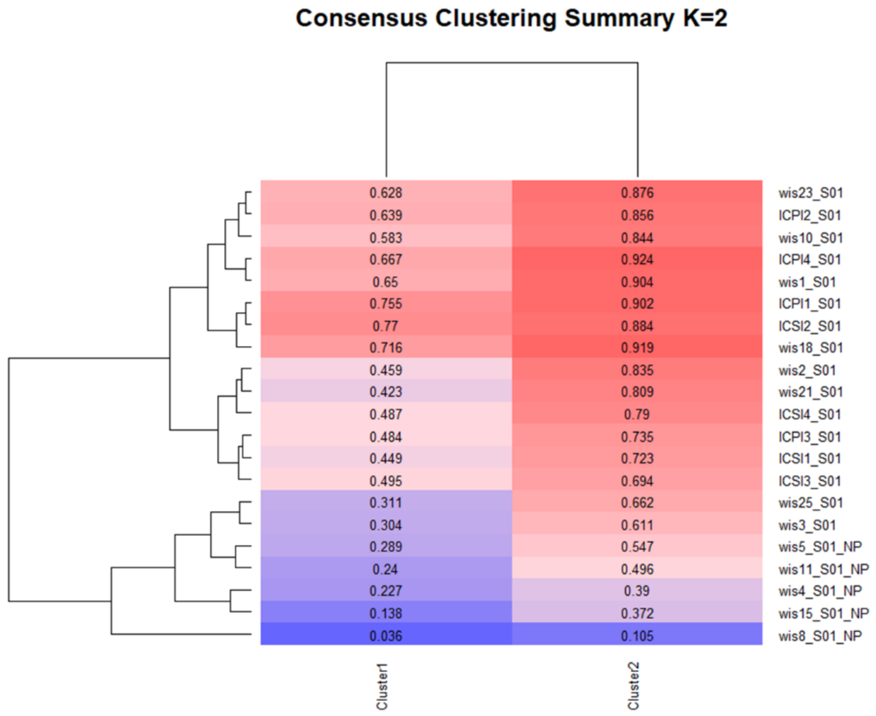

Mean Consensus Scores

K	C1	C2	C3	C4
2	0.972	0.99	NA	NA
3	0.803	0.819	0.91	NA
4	0.743	0.77	0.7	0.82

PAC Scores

K	PAC
2	0.072
3	0.400
4	0.351
5	0.236
6	0.216
7	0.237
8	0.236
9	0.229
10	0.217

Unsupervised consensus clustering (CC) algorithm (ConsensusClusterPlus) in R (version 3.4.1)[1] was applied to 16 pain, urgency and frequency symptoms together with five non-pelvic Wisconsin Symptom Survey (WSS) questions from K=2 to 10 clusters. Mean consensus scores[2] and proportion of ambiguously clustered pairs (PAC)[5] were used to determine optimal number of clusters. The optimal number of clusters K was chosen to be the number of clusters with the highest mean consensus score within clusters and lowest PAC scores. Both mean consensus and PAC tables are included in the above figure. The two-cluster solution, K=2, was chosen to be the optimal number of clusters. Given the optimal number of clusters, K=2, the mean values of the 21 scaled symptoms were calculated within cluster and are summarized in the heatmap. Cooler colors indicate mean scores close to 0 while warmer scores indicate mean scores closer to 1. Symptoms with the NP tag indicate the non-pelvic WSS score questions. A legend key for the 21 items summarized in the heatmap can be found in Extended Figure 2.

Extended Figure 2. Legend Key for the 21 items used in Consensus Clustering

Scaled Variable	Description
wis23_S01	Urgency to Urinate
ICPI2_S01	Getting up at night to urinate?
wis10_S01	Getting Up at Night to Go to the Bathroom
ICPI4_S01	Burning, pain, discomfort, or pressure in your bladder?
wis1_S01	Bladder Discomfort
ICPI1_S01	Frequent Urination during the day?
ICSI2_S01	During the past month, have you had to urinate less than 2 hours after you finished urinating?
wis18_S01	Going to the Bathroom frequently during the day
wis2_S01	Bladder Pain
wis21_S01	Difficulty Sleeping because of Bladder Symptoms
ICSI4_S01	During the past month, have you experienced pain or burning in your bladder?
ICPI3_S01	Need to urinate with little warning?
ICSI1_S01	During the past month, how often have you felt the strong need to urinate with little or no warning?
ICSI3_S01	During the past month, how often did you most typically get up at night to urinate?
wis25_S01	Burning Sensation in Bladder
wis3_S01	Other Pelvic Discomfort
wis5_S01_NP	Backache (Non-pelvic WSS)
wis11_S01_NP	Aches in Joints (Non-pelvic WSS)
wis4_S01_NP	Headache (Non-pelvic WSS)
wis15_S01_NP	Abdominal Cramps (Non-pelvic WSS)
wis8_S01_NP	Chest Pain (Non-pelvic WSS)

Extended Figure 3. Prediction of membership to high widespreadness consensus cluster (Cluster 2) given sum of non-pelvic WSS items with severity ≥2

wide5_2_x	estimated prob.	sensitivity	specificity	sum
0	0.2198	1.0000	0.0000	1.0000
1	0.3617	0.9348	0.2365	1.1713
2	0.5326	0.7705	0.5676	1.3381
3	0.6962	0.6034	0.8007	1.4041
4	0.8217	0.3201	0.9426	1.2627
5	0.9026	0.0652	0.9831	1.0483

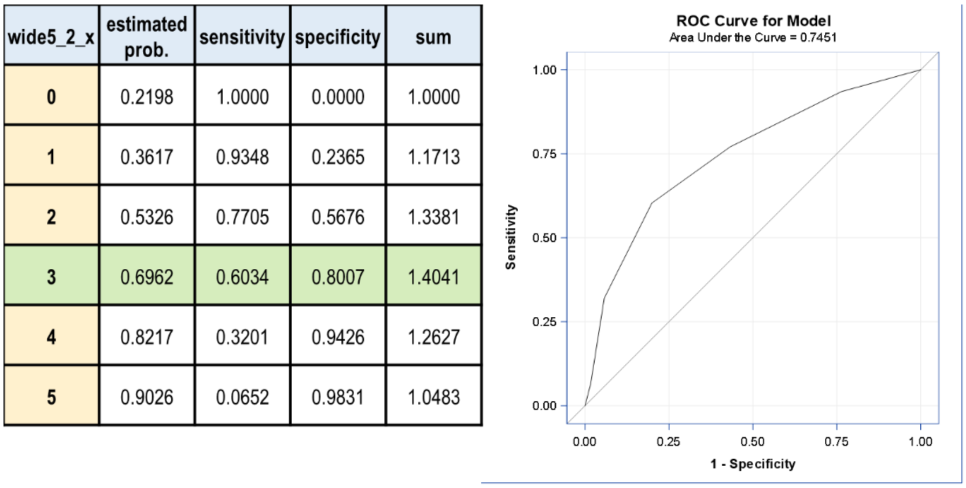

A logistic regression model was fit to predict membership into consensus cluster 2 given the sum of non-pelvic WSS items (0-5) with a severity greater than or equal to 2 as predictor. The sensitivity and specificity of the potential cutoffs are displayed in both the ROC table and plot. The cutoff with the highest sum was chosen to be the criteria for membership into the high widespreadness of pain subgroup. From these results, patients with three or more non-pelvic WSS items with a severity greater than or equal to 2 were classified as belonging to cluster 2. The area under the curve indicating the strength of the predictor in modeling of the outcome is included in the ROC curve plot.

Extended Figure 4. Prediction of membership to high widespreadness consensus cluster (Cluster 2) given the maximum severity level of non-pelvic WSS items for three or more items

wide5_x_3	estimated prob.	sensitivity	specificity	sum
0	0.3111	1.0000	0.0000	1.0000
1	0.4788	0.7620	0.5405	1.3026
2	0.6514	0.6034	0.8007	1.4041
3	0.7918	0.4306	0.9324	1.3630
4	0.8855	0.2578	0.9831	1.2409
5	0.9402	0.1303	0.9966	1.1269
6	0.9697	0.0482	1.0000	1.0482

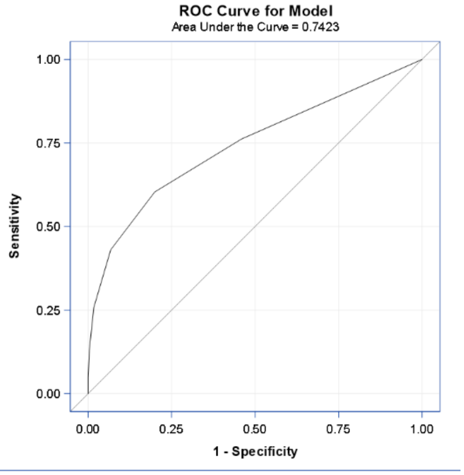

A logistic regression model was fit to predict membership into consensus cluster 2 given the maximum severity (0-6) where three or more of the non-pelvic WSS items are greater than or equal to the severity level as predictor. The sensitivity and specificity of the potential cutoffs are displayed in both the ROC table and plot. The cutoff with the highest sum was chosen to be the criteria for membership into the high widespreadness of pain subgroup. From these results, patients with a severity threshold of 2 or higher for three or more items were classified as belonging to cluster 2. The area under the curve indicating the strength of the predictor in modeling of the outcome is included in the ROC curve plot.

Extended Figure 5. Primary analysis modeling change in pelvic pain (on NRS scale) at end of study from average baseline

Model Results

Oral PPS and Hydroxyzine

Coefficients (Change in Pain from Baseline)	Estimate	Standard Error	95% CI Lower Limit	95% CI Upper Limit	T Score	p-value
Intercept	2.36	1.22	-0.04	4.76	1.93	0.054
Avg. Baseline Pain	-0.27	0.15	-0.57	0.02	-1.81	0.070
Female	-0.67	0.57	-1.79	0.46	-1.17	0.244
Age	-0.01	0.01	-0.04	0.01	-1.12	0.263
Caucasian	-0.04	0.52	-1.06	0.97	-0.08	0.936
Widespread Pain[a]	-0.19	0.44	-1.06	0.68	-0.43	0.664
Trt. Eff. High Wide[b]	-0.16	0.62	-1.37	1.04	-0.27	0.790
Trt. Eff. Low Wide[b]	-1.44	0.61	-2.64	-0.25	-2.37	0.018

[a] High vs. Low Widespread Pain

[b] PPS/Hydrox vs. Placebo or Single Trt. within Widespread Pain Sub.

Intravesical BCG

Coefficients (Change in Pain from Baseline)	Estimate	Standard Error	95% CI Lower Limit	95% CI Upper Limit	T Score	p-value
Intercept	0.62	1.14	-1.61	2.86	0.55	0.584
Avg. Baseline Pain	-0.20	0.12	-0.43	0.04	-1.65	0.099
Female	-0.24	0.39	-1.01	0.52	-0.62	0.534
Age	0.00	0.01	-0.02	0.02	-0.16	0.871
Caucasian	-0.03	0.44	-0.89	0.83	-0.07	0.948
Widespread Pain[a]	0.26	0.42	-0.56	1.08	0.62	0.535
Trt. Eff. High Wide[b]	-0.54	0.42	-1.36	0.28	-1.30	0.195
Trt. Eff. Low Wide[b]	-0.40	0.40	-1.18	0.38	-1.01	0.311

[a] High vs. Low Widespread Pain

[b] BCG vs. Placebo within Widespread Pain Subgroup

Oral Amitriptyline with EBMP

Coefficients (Change in Pain from Baseline)	Estimate	Standard Error	95% CI Lower Limit	95% CI Upper Limit	T Score	p-value
Intercept	0.39	0.76	-1.10	1.87	0.51	0.607
Avg. Baseline Pain	-0.61	0.09	-0.78	-0.43	-6.68	<0.001
Female	0.09	0.40	-0.70	0.89	0.24	0.814
Age	0.00	0.01	-0.02	0.02	-0.15	0.883
Caucasian	0.37	0.33	-0.28	1.01	1.12	0.263
Widespread Pain[a]	1.87	0.44	1.00	2.73	4.24	<0.001
Trt. Eff. High Wide[b]	-1.14	0.48	-2.08	-0.19	-2.35	0.019
Trt. Eff. Low Wide[b]	0.05	0.36	-0.66	0.75	0.14	0.892

[a] High vs. Low Widespread Pain

[b] Amitriptyline+EBMP vs. Placebo+EBMP within Widespread Pain Sub.

Estimated Mean Trajectory

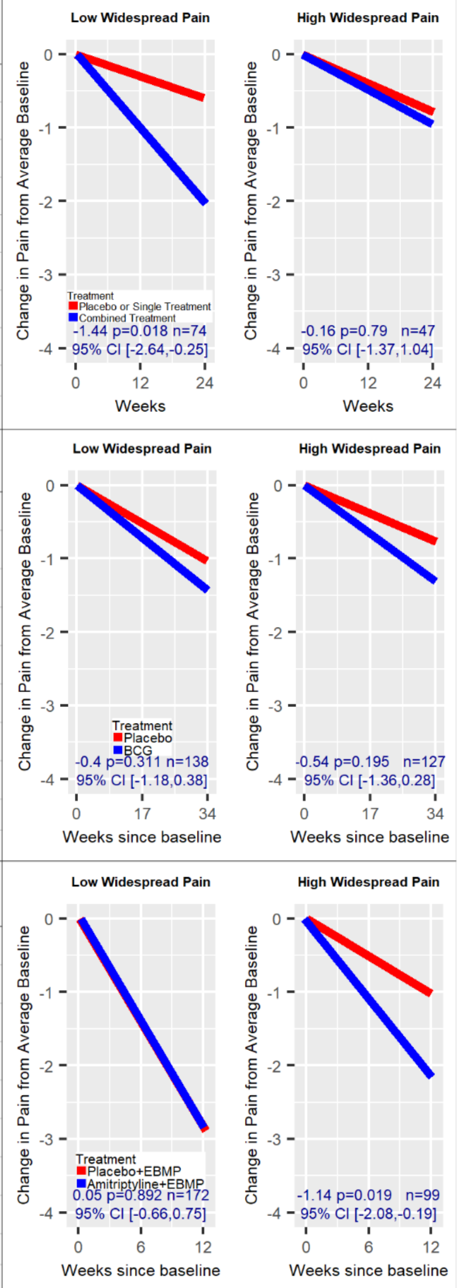

Table on left are the results for the primary analysis modeling change in pelvic pain at end of study from average baseline by widespreadness subgroup. Multiple imputation modeling was utilized to impute all missing outcomes and baseline covariates. Mean trajectory plots on the right were derived from the model coefficients contained in the table and represent the estimated change in outcome from average baseline at the end of the study for the average participant within RCT accounting for baseline outcome, age, race, and sex. P-values within plot test the difference between treatment and control and were derived from the primary analysis results contained in the table.

Extended Figure 6. Primary analysis modeling change in urinary urgency (on NRS scale) at end of study from average baseline

Model Results

Oral PPS and Hydroxyzine

Coefficients (Change in Urg. from Baseline)	Estimate	Standard Error	95% CI Lower Limit	95% CI Upper Limit	T Score	p-value
Intercept	2.16	1.00	0.21	4.11	2.17	0.030
Avg. Baseline Pain	-0.23	0.11	-0.45	-0.01	-2.04	0.041
Female	-0.97	0.49	-1.93	-0.01	-1.98	0.048
Age	-0.01	0.01	-0.03	0.01	-1.23	0.219
Caucasian	-0.14	0.44	-1.00	0.72	-0.31	0.755
Widespread Pain[a]	0.13	0.37	-0.59	0.86	0.36	0.718
Trt. Eff. High Wide[b]	0.07	0.53	-0.96	1.11	0.14	0.889
Trt. Eff. Low Wide[b]	-1.09	0.51	-2.08	-0.10	-2.15	0.031

[a] High vs. Low Widespread Pain

[b] PPS/Hydrox vs. Placebo or Single Trt. within Widespread Pain Sub.

Intravesical BCG

Coefficients (Change in Urg. from Baseline)	Estimate	Standard Error	95% CI Lower Limit	95% CI Upper Limit	T Score	p-value
Intercept	0.95	1.01	-1.02	2.92	0.94	0.345
Avg. Baseline Pain	-0.31	0.11	-0.52	-0.10	-2.94	0.003
Female	0.04	0.36	-0.67	0.75	0.11	0.916
Age	0.01	0.01	-0.01	0.03	0.74	0.461
Caucasian	-0.24	0.41	-1.04	0.56	-0.59	0.553
Widespread Pain[a]	0.25	0.39	-0.51	1.00	0.65	0.518
Trt. Eff. High Wide[b]	-0.52	0.39	-1.28	0.24	-1.33	0.183
Trt. Eff. Low Wide[b]	-0.60	0.37	-1.32	0.12	-1.63	0.103

[a] High vs. Low Widespread Pain

[b] BCG vs. Placebo within Widespread Pain Subgroup

Oral Amitriptyline with EBMP

Coefficients (Change in Urg. from Baseline)	Estimate	Standard Error	95% CI Lower Limit	95% CI Upper Limit	T Score	p-value
Intercept	0.97	0.79	-0.58	2.52	1.23	0.219
Avg. Baseline Pain	-0.68	0.08	-0.84	-0.52	-8.34	<0.001
Female	-0.03	0.40	-0.81	0.75	-0.09	0.932
Age	0.00	0.01	-0.02	0.02	-0.13	0.898
Caucasian	0.42	0.33	-0.22	1.06	1.29	0.196
Widespread Pain[a]	1.50	0.43	0.66	2.34	3.50	0.001
Trt. Eff. High Wide[b]	-0.80	0.49	-1.75	0.16	-1.63	0.103
Trt. Eff. Low Wide[b]	-0.07	0.36	-0.78	0.63	-0.21	0.835

[a] High vs. Low Widespread Pain

[b] Amitriptyline+EBMP vs. Placebo+EBMP within Widespread Pain Sub.

Estimated Mean Trajectory

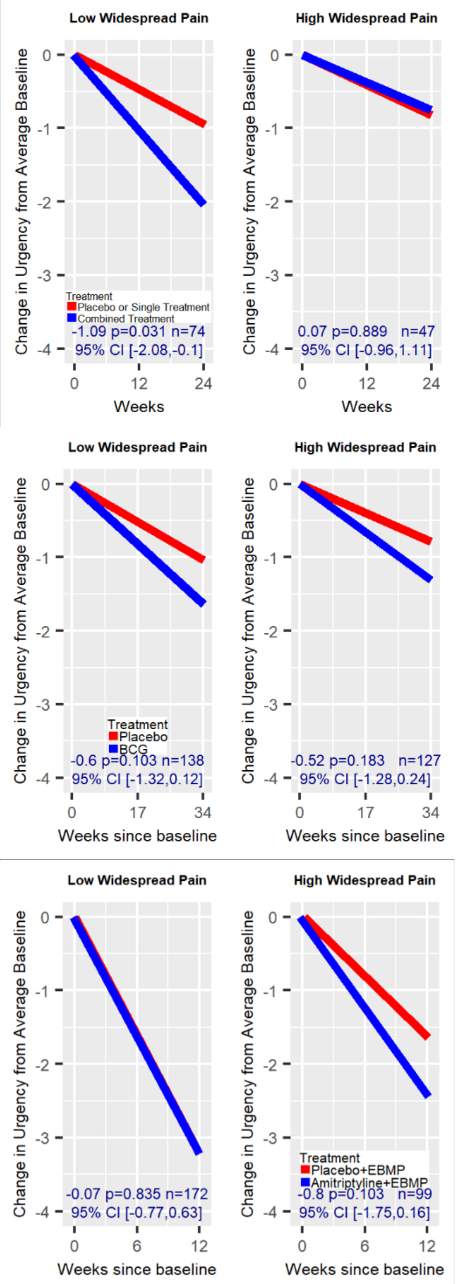

Table on left are the results for the primary analysis modeling change in urinary urgency at end of study from average baseline by widespreadness subgroup. Multiple imputation modeling was utilized to impute all missing outcomes and baseline covariates. Mean trajectory plots on the right were derived from the model coefficients contained in the table and represent the estimated change in outcome from average baseline at the end of the study for the average participant within RCT accounting for baseline outcome, age, race, and sex. P-values within plot test the difference between treatment and control and were derived from the primary analysis results contained in the table.

Extended Figure 7. Primary analysis modeling change in urinary frequency (on NRS scale) at end of study from average baseline

Model Results

Oral Amitriptyline with EBMP

Coefficients (Change in Freq. from Baseline)	Estimate	Standard Error	95% CI Lower Limit	95% CI Upper Limit	T Score	p-value
Intercept	-0.21	0.87	-1.92	1.50	-0.24	0.812
Avg. Baseline Pain	-0.49	0.09	-0.66	-0.32	-5.55	<0.001
Female	-0.32	0.41	-1.12	0.47	-0.79	0.428
Age	0.01	0.01	-0.02	0.03	0.51	0.611
Caucasian	0.39	0.34	-0.28	1.05	1.14	0.254
Widespread Pain[a]	1.70	0.44	0.83	2.57	3.83	<0.001
Trt. Eff. High Wide[b]	-1.53	0.49	-2.50	-0.56	-3.10	0.002
Trt. Eff. Low Wide[b]	-0.24	0.37	-0.96	0.48	-0.65	0.514

[a] High vs. Low Widespread Pain

[b] Amitriptyline+EBMP vs. Placebo+EBMP within Widespread Pain Subgroup

Estimated Mean Trajectory

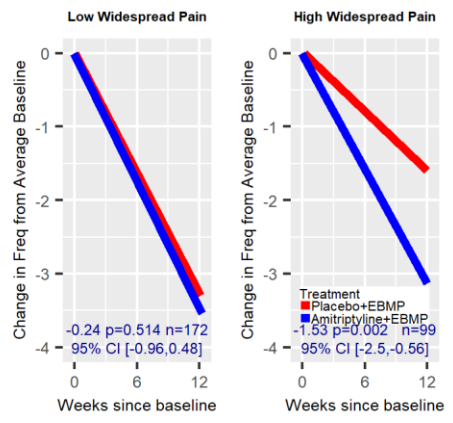

Table on left are the results for the primary analysis modeling change in urinary frequency at end of study from average baseline by widespreadness subgroup for the amitriptyline trial. Multiple imputation modeling was utilized to impute all missing outcomes and baseline covariates. Mean trajectory plots on the right were derived from the model coefficients contained in the table and represent the estimated change in outcome from average baseline at the end of the study for the average participant within RCT accounting for baseline outcome, age, race, and sex. P-values within plot test the difference between treatment and control and were derived from the primary analysis results contained in the table.

Extended Figure 8. 1-ECDF plots and corresponding tables for the proportion of patients with observed percentage improvement in pelvic pain beyond average baseline

Observed Percentage Change

Oral PPS and Hydroxyzine

Observed Proportion of Improvers at or Above	Widespread Pain Subgroup	Combination Treatment	Placebo or Single Treatment	Difference	NNT
30% Improvement from Baseline	Low	0.47	0.21	0.26	3.84
	High	0.31	0.21	0.10	9.82
50% Improvement from Baseline	Low	0.35	0.09	0.27	3.77
	High	0.15	0.18	-0.02	-44.05
		p-value			
Wilcoxon Rank Sum Test: Difference in Observed Improvement Between Treatments	Low	0.0803			
	High	0.9329			

Intravesical BCG

Observed Proportion of Improvers at or Above	Widespread Pain Subgroup	BCG	Placebo	Difference	NNT
30% Improvement from Baseline	Low	0.34	0.25	0.08	11.86
	High	0.25	0.27	-0.02	-53.48
50% Improvement from Baseline	Low	0.27	0.21	0.06	17.06
	High	0.18	0.16	0.02	52.36
		p-value			
Wilcoxon Rank Sum Test: Difference in Observed Improvement Between Treatments	Low	0.3659			
	High	0.14			

Oral Amitriptyline with EBMP

Observed Proportion of Improvers at or Above	Widespread Pain Subgroup	Amitriptyline + EBMP	Placebo + EBMP	Difference	NNT
30% Improvement from Baseline	Low	0.58	0.63	-0.05	-21.28
	High	0.47	0.37	0.10	9.74
50% Improvement from Baseline	Low	0.45	0.46	0.00	-303.03
	High	0.43	0.19	0.23	4.29
		p-value			
Wilcoxon Rank Sum Test: Difference in Observed Improvement Between Treatments	Low	0.9678			
	High	0.0999			

1-ECDF Plots

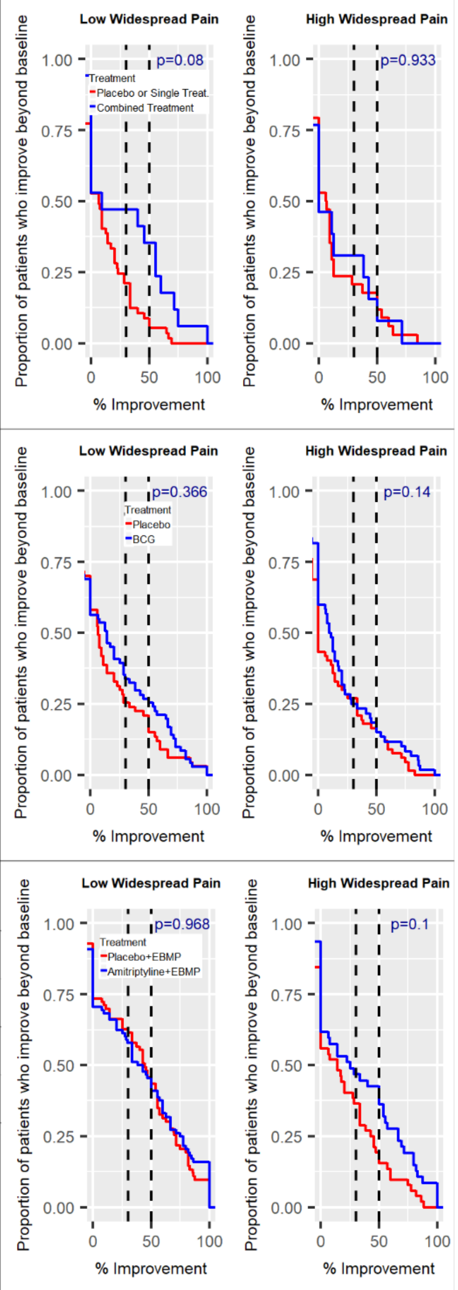

The 1-ECDF plots (right within cell) represent the proportion of patients whose percentage improvement in pelvic pain is beyond average baseline at the end of study given the observed data within respective widespreadness subgroup. Patients missing end of study data have an observed percentage change of 0. P-value for testing the difference in observed percentage improvement curves for treatment (Blue) and control (Red) are displayed within plot and tables and were derived from the non-parametric Wilcoxon Rank-Sum test. The tables to the left of the plots correspond to the 1-ECDF curves and summarize the proportion of patients who have at least 30% and 50% improvement from baseline. Differences between proportion of improvement between treatment and control and the Number Needed to Treat (NNT) were calculated for each percentage improvement benchmark.

Extended Figure 9. 1-ECDF plots and corresponding tables for the proportion of patients with observed percentage improvement in urinary urgency beyond average baseline

Observed Percentage Change

Oral PPS and Hydroxyzine

Observed Proportion of Improvers at or Above	Widespread Pain Subgroup	Combination Treatment	Placebo or Single Treatment	Difference	NNT
30% Improvement from Baseline	Low	0.29	0.18	0.12	8.42
	High	0.08	0.12	-0.04	-24.57
50% Improvement from Baseline	Low	0.29	0.05	0.24	4.14
	High	0.00	0.12	-0.12	-8.50
		p-value			
Wilcoxon Rank Sum Test: Difference in Observed Improvement Between Treatments	Low	0.3081			
	High	0.7093			

Intravesical BCG

Observed Proportion of Improvers at or Above	Widespread Pain Subgroup	BCG	Placebo	Difference	NNT
30% Improvement from Baseline	Low	0.37	0.27	0.10	10.26
	High	0.27	0.24	0.03	35.84
50% Improvement from Baseline	Low	0.23	0.16	0.06	16.34
	High	0.18	0.10	0.08	12.69
		p-value			
Wilcoxon Rank Sum Test: Difference in Observed Improvement Between Treatments	Low	0.1952			
	High	0.1132			

Oral Amitriptyline with EBMP

Observed Proportion of Improvers at or Above	Widespread Pain Subgroup	Amitriptyline + EBMP	Placebo + EBMP	Difference	NNT
30% Improvement from Baseline	Low	0.66	0.58	0.08	12.38
	High	0.45	0.37	0.08	12.29
50% Improvement from Baseline	Low	0.50	0.43	0.07	15.08
	High	0.36	0.25	0.11	8.95
		p-value			
Wilcoxon Rank Sum Test: Difference in Observed Improvement Between Treatments	Low	0.8003			
	High	0.0853			

1-ECDF Plots

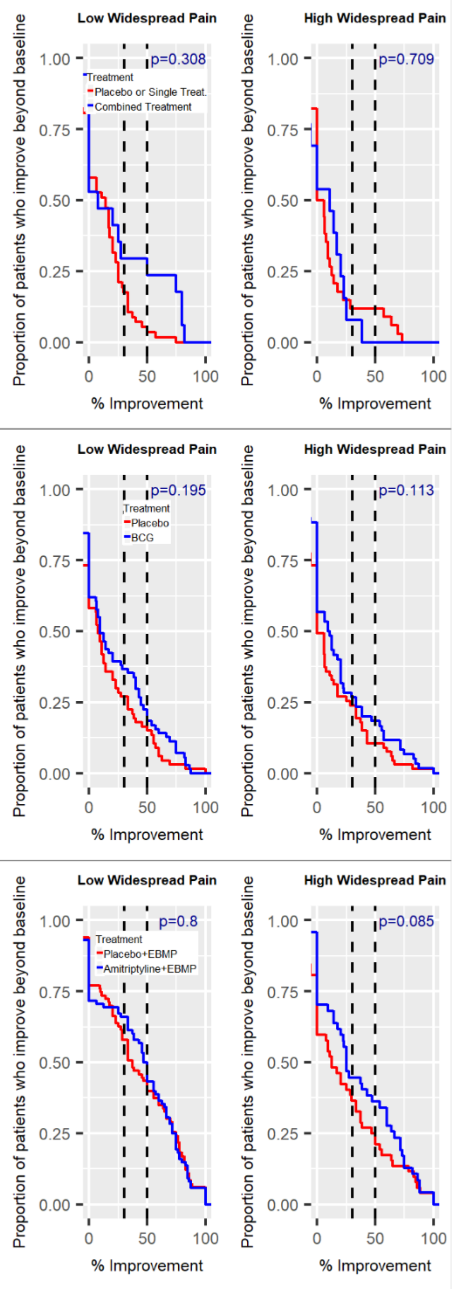

The 1-ECDF plots (right within cell) represent the proportion of patients whose percentage improvement in urinary urgency is beyond average baseline at the end of study given the observed data within respective widespreadness subgroup. Patients missing end of study data have an observed percentage change of 0. P-value for testing the difference in observed percentage improvement curves for treatment (Blue) and control (Red) are displayed within plot and tables and were derived from the non-parametric Wilcoxon Rank-Sum test. The tables to the left of the plots correspond to the 1-ECDF curves and summarize the proportion of patients who have at least 30% and 50% improvement from baseline. Differences between proportion of improvement between treatment and control and the Number Needed to Treat (NNT) were calculated for each percentage improvement benchmark.

Extended Figure 10. 1-ECDF plots and corresponding tables for the proportion of patients with observed percentage improvement in urinary frequency beyond average baseline

Oral Amitriptyline with EBMP

Observed Percentage Change

Observed Proportion of Improvers at or Above	Widespread Pain Subgroup	Amitriptyline + EBMP	Placebo + EBMP	Difference	NNT
30% Improvement from Baseline	Low	0.65	0.64	0.01	109.89
	High	0.53	0.33	0.21	4.88
50% Improvement from Baseline	Low	0.53	0.48	0.05	19.16
	High	0.43	0.19	0.23	4.29

		p-value
Wilcoxon Rank Sum Test: Difference in Observed Improvement Between Treatments	Low	0.4204
	High	0.0168

1-ECDF Results

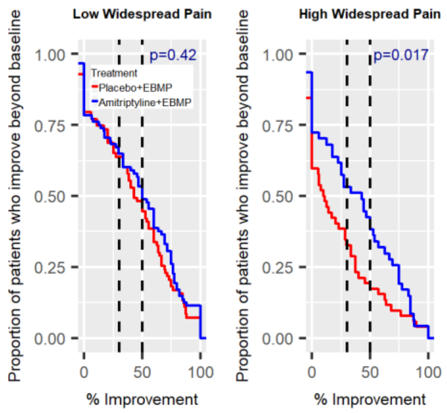

The 1-ECDF plots (right within cell) represent the proportion of patients whose percentage improvement in urinary frequency is beyond average baseline at the end of study given the observed data within respective widespreadness subgroup for the amitriptyline trial. Patients missing end of study data have an observed percentage change of 0. P-value for testing the difference in observed percentage improvement curves for treatment (Blue) and control (Red) are displayed within plot and tables and were derived from the non-parametric Wilcoxon Rank-Sum test. The tables to the left of the plots correspond to the 1-ECDF curves and summarize the proportion of patients who have at least 30% and 50% improvement from baseline. Differences between proportion of improvement between treatment and control and the Number Needed to Treat (NNT) were calculated for each percentage improvement benchmark.

Supplementary Online Content

These materials describe our use of standard statistical approaches to the specific methods described and provide limited results specific to these methods and tables reflecting those methods. They are provided in case the reader has an interest in the specifics of how e applied these standard techniques.

Supplementary Methods 12
Consensus Clustering (CC) *12*
Determining the High Widespreadness of Pain Subgroup Criteria *12*
Multiple Imputation Models *12*
Mean Trajectory Plots *13*
1-ECDF Empirical Cumulative Distribution Functions Plots *13*
Supplementary Results 13
Consensus Clustering *13*
Optimizing the Widespreadness Subgroup Criteria with Consensus Clustering Results *13*
Supplementary Tables 14
Supplementary Table 1. Observed change in outcome and estimated treatment effects for complete case and multiple imputation models *14*
Supplementary Table 2. Observed change in outcome and estimated treatment effects for complete case and multiple imputation models by widespreadness subgroup *15*
Supplementary Table 3. Estimated change in NRS measure at end of study from average baseline for the average RCT participant *17*
Supplementary References 18

Supplementary Methods

Consensus Clustering (CC)

ConsensusClusterPlus in R (version 3.4.1) was utilized to form subgroups of patients with similar baseline responses of pelvic pain, urgency, frequency and non-pelvic pain in the combined PPS/hydroxyzine, BCG and amitriptyline dataset.[1,2] Twenty-one baseline symptoms were included as inputs in the consensus clustering (CC) including four from the Interstitial Cystitis Problem Index (ICPI) survey[3], four from the Interstitial Cystitis Symptom Index (ICSI) survey[3], eight from the Wisconsin Symptom Survey (WSS) indicating severity of pelvic pain and urinary symptoms, and five for the WSS indicating severity of non-pelvic pain symptoms.[4] Each of the 21 items were scaled between 0 and 1 by dividing by the maximum value on the scale. One thousand replications of k-means clustering with 80% patient resampling was utilized in the CC algorithm given the scaled data from N=649 patients. The algorithm was run from k=2 through k=10 clusters to identify the "optimal" number of clusters, K, in the dataset. To identify K, we utilized the proportion of ambiguously clustered pairs (PAC) and mean consensus scores to make a determination.[1,2,5] The k with the lowest PAC and highest mean consensus scores within cluster was chosen to be optimal.

After the optimal K was identified, the mean of each of the scaled 21 items was calculated within cluster and displayed in a heatmap. The cells of the heatmap represents the average value of the scaled symptom within cluster where blue cells have a value close to 0 and red cells close to 1. The heatmap.2 function from the gplots package in R was used to form the heatmap.[6] A summary of CC results are presented in Extended Figures 1-2.

Determining the High Widespreadness of Pain Subgroup Criteria

Since clustering results are sample dependent, the optimal number of clusters K, and/or the severity of symptoms within clusters will vary for new RCTs. Thus, a highly reliable classification rule will be needed to guide baseline stratification into high/low pain widespreadness subgroups in advance of baseline data collection for new RCT studies. The CC results obtained from the harmonized baseline dataset (across 3 RCTs) resulted in K=2 clusters (with high mean consensus for both clusters), where Cluster 1 patients were indicative of low pain widespreadness, as the five non pelvic WSS severity was lower, and Cluster 2 of high pain widespreadness, as the five non-pelvic WSS severity was higher (eFigures 1-2).

We developed a criterion for membership in the high widespreadness of pain cluster, Cluster 2, given the count and severity of five non-pelvic WSS questions. Predicted membership in Cluster 2 was modeled with a logistic regression model. In two separate univariate models we include as a predictor the number of five non-pelvic WSS items with a severity ≥2/6. Given the results of these models, a classification rule of three or more of the 5 WSS items with levels ≥2/6 was selected to have optimal ROC characteristic. In both models, a ROC curve is plotted along with a table of sensitivity and specificity values for each of the possible cutoffs of the predictor. The AUC was also calculated to determine the strength of the predictor in modeling the outcome of interest.[7] The cutpoint of the predictor with the highest sum of sensitivity and severity, which if subtracted from 1 would also be known as the Youden index, was chosen to be the optimal cutpoint.[8] ROC curve analyses and sensitivity and specificity tables for both models are shown in Extended Figures 3-4.

Multiple Imputation Models

Missing data in each of the three RCTs precludes a conventional intent-to-treat (ITT) analysis without imputation. We utilized multiple imputation with predictive mean matching, m=100 imputations and K=5 closest observations, to impute all missing intermediate and end of study numeric rating scale (NRS) measures of pelvic pain, urinary urgency and frequency as well as the GRA outcome on the 1-7 ordinal scale.[9-11] Each imputation model was run separately for pelvic pain, urinary urgency, frequency and GRA outcomes within each RCT. The imputation model included all longitudinal measures of the outcome of interest, the longitudinal GRA assessment, treatment assignment, widespreadness subgroup indicator and baseline measures of age, race, and sex. In the sensitivity analyses where we model treatment response without regards to widespreadness subgroup, the widespreadness indicator was not included in the imputation model.

Absolute change in NRS outcome at the end of the study for pelvic pain, urinary urgency, and frequency outcomes and the GRA response were calculated after the outcome was imputed for each of the m=100 datasets. Rubin's rules were used to estimate the pooled coefficients and standard errors from the models run across all imputed datasets.[11]

Mean Trajectory Plots

The mean trajectory plots as displayed in Figures 1-3 and Extended Figures 5-7 represent the estimated change in NRS outcome from baseline at the end of the study within treatment arm and widespreadness subgroup for the average RCT participant. They are derived from linear combinations of model coefficients from the primary analysis using multiple imputation. Coefficients for the primary analysis models are included in the tables to the left of the plots in Extended Figures 5-7. A table containing the estimated change in NRS outcome at the end of study for each treatment and widespreadness group along with 95% confidence intervals are contained in Supplementary Table 3. The change in outcome is calculated for the average RCT participant (See Supplementary Table 3 footnotes).

1-ECDF Empirical Cumulative Distribution Functions Plots

We calculated the observed percentage change in outcome at the end of study from baseline for all NRS outcomes from the following formula.

$$\%\, Improvement\ from\ Baseline = \frac{Baseline\ Value - End\ of\ Study\ Value}{Baseline\ Value} x100 \qquad (S1)$$

For RCT participants who were missing end of study outcome, we assumed their percentage improvement was 0. The ggplot2 package in R [12] was used to calculate the 1-ECDF (Empirical cumulative distribution functions) plot for all treatment arm and widespreadness subgroup combinations. The y axis represents the proportion of patients who improve beyond the percentage indicated on the x-axis. In addition, we calculate the proportion of patients who have 30% improvement or higher and 50% improvement or higher in outcome from baseline within treatment and widespreadness combination which are displayed in the tables in Extended Figures 8-10. The difference in proportion of improvers is calculated between treatment and control for each widespreadness subgroup as well as the Number Needed to Treat (NNT).[13] The non-parametric two-sided Wilcoxon Rank Sum test utilizing normal approximation was used to test the difference in observed percentage change improvement curves between treatment and control within widespreadness subgroup.[14]

Supplementary Results

Consensus Clustering

From the CC algorithm we determined that K=2 clusters was optimal, having the lowest PAC of 0.072 across all K and the highest mean consensus scores within cluster of 0.972 and 0.990 for Cluster 1 and Cluster 2 respectively (Figure S9). Between the two clusters, there was a strong separation in mean severity of the urinary pain, urgency and frequency items that tracked with the differential prevalence of the five scaled non-pelvic WSS questions of Headache (C1: 0.227, C2: 0.390), Backache (C1: 0.289, C2: 0.547), Chest Pain (C1: 0.036, C2: 0.105), Ache in Joints (C1: 0.240, C2: 0.496), and Abdominal Cramps (C1: 0.138, C2: 0.372). We determined that patients belonging to Cluster 1 are indicative of a low widespreadness of pain subgroup and Cluster 2 as a high widespreadness of pain subgroup (eFigures 1-2).

Optimizing the Widespreadness Subgroup Criteria with Consensus Clustering Results

In the univariate logistic regression model with the sum of WSS items exceeding a symptom severity threshold of two or greater as predictor, we found that the cutoff of three or more of the five items provided the highest Youden index of 0.404 with a model AUC of 0.745 (eFigure 3). For the logistic regression model with the maximum severity (0-6) where three or more of the non-pelvic WSS items are greater than or equal to the severity level as predictor, we verify that a severity threshold of two or higher is optimal with a Youden index of 0.404 and model AUC of 0.742 (eFigure 4). From these models, we determine that a symptom severity score of two or higher for three or more items indicates a patient belongs to the high widespreadness of pain subgroup. We used this criterion to indicate the high widespreadness of pain subgroup in our article.

Supplementary Tables

Supplementary Table 1. Observed change in outcome and estimated treatment effects for complete case and multiple imputation models

	Complete Cases			Intent to Treat
	**Observed Change: Control mean (SD)**	**Observed Change: Treatment mean (SD)**	**Complete Case Model: Treatment[c]**	**MI Model: Treatment[c]**
**Oral PPS and Hydroxyzine**				
**N**	74	23	97[d]	121[d]
Logistic Regression on Log Odds Scale for Binary Outcome				
**GRA Responder[a]**	19 (26%)	12 (52%)	1.18 (0.02) [0.19,2.17][e]	1.09 (0.027) [0.12,2.06][e]
Absolute Change on NRS Scale				
**Change in Pain NRS[b]**	-0.76 (1.79)	-1.63 (1.95)	-0.97 (0.029) [-1.85,-0.10]	-0.83 (0.052) [-1.66,0.01]
**Change in Urgency NRS[b]**	-0.96 (1.61)	-1.28 (1.56)	-0.52 (0.184) [-1.28,0.25]	-0.55 (0.134) [-1.26,0.17]
**Intravesical BCG**				
**N**	126	120	246[d]	265[d]
Logistic Regression on Log Odds Scale for Binary Outcome				
**GRA Responder[a]**	16 (13%)	27 (23%)	0.71 (0.037) [0.04,1.37][e]	0.67 (0.048) [0.01,1.33][e]
Absolute Change on NRS Scale				
**Change in Pain NRS[b]**	-0.91 (2.12)	-1.44 (2.43)	-0.51 (0.086) [-1.09,0.07]	-0.49 (0.087) [-1.06,0.07]
**Change in Urgency NRS[b]**	-0.93 (1.99)	-1.53 (2.32)	-0.63 (0.024) [-1.17,-0.08]	-0.57 (0.034) [-1.09,-0.04]
**Oral Amitriptyline with EBMP**				
**N**	119	111	230[d]	270[d]
Logistic Regression on Log Odds Scale for Binary Outcome				
**GRA Responder[a]**	61 (51%)	74 (67%)	0.66 (0.017) [0.12,1.20][e]	0.67 (0.012) [0.15,1.19][e]
Absolute Change on NRS Scale				
**Change in Pain NRS[b]**	-2.25 (2.42)	-2.62 (2.49)	-0.48 (0.116) [-1.08,0.12]	-0.46 (0.128) [-1.05,0.13]
**Change in Urgency NRS[b]**	-2.51 (2.56)	-3.05 (2.50)	-0.50 (0.098) [-1.09,0.09]	-0.47 (0.112) [-1.04,0.11]
**Change in Frequency NRS[b]**	-2.61 (2.51)	-3.51 (2.28)	-0.85 (0.006) [-1.46,-0.25]	-0.78 (0.011) [-1.38,-0.18]

[a] No. (%)

[b] Change is calculated as the outcome at the end of study subtracted from averaged baseline

[c] Estimated treatment effect (Change in outcome on treatment compared to control within widespreadness subgroup) from model results: Effect (p-value) [95% CI]

[d] Sample size of treatment and control within widespreadness subgroup

[e] Treatment effect log odds ratio from model results: Log odds ratio (p-value) [95% CI]

Supplementary Table 2. Observed change in outcome and estimated treatment effects for complete case and multiple imputation models by widespreadness subgroup

	Low Widespreadness				High Widespreadness				
	Complete Cases			Intent to Treat	Complete Cases			Intent to Treat	
	Observed Change: Control mean (SD)	Observed Change: Treatment mean (SD)	Complete Case Model: Treatment[c]	MI Model: Treatment[c]	Observed Change: Control mean (SD)	Observed Change: Treatment mean (SD)	Complete Case Model: Treatment[c]	MI Model: Treatment[c]	MI Model: Treatment Interaction[d]
**Oral PPS and Hydroxyzine**									
**N**	47	11	58[e]	74[e]	27	12	39[e]	47[e]	
Logistic Regression on Log Odds Scale for Binary Outcome									
**GRA Responder[a]**	11 (23%)	7 (64%)	1.70 (0.019) [0.28,3.11][f]	1.28 (0.059) [-0.05,2.61][f]	8 (30%)	5 (42%)	0.63 (0.392) [-0.81,2.07][f]	0.64 (0.379) [-0.79,2.07][f]	-0.64 (0.522) [-2.60,1.32]
Absolute Change on NRS Scale									
**Change in Pain NRS[b]**	-0.68 (1.77)	-2.36 (1.87)	-1.78 (0.005) [-3.00,-0.57]	-1.44 (0.018) [-2.64,-.25]	-0.91 (1.84)	-0.96 (1.84)	-0.20 (0.755) [-1.45,1.05]	-0.16 (0.790) [-1.37,1.04]	1.28 (0.141) [-0.42,2.99]
**Change in Urgency NRS[b]**	-1.05 (1.48)	-2.05 (1.74)	-1.11 (0.038) [-2.16,-0.06]	-1.09 (0.031) [-2.08,-0.10]	-0.80 (1.83)	-0.58 (1.00)	-0.02 (0.970) [-1.09,1.05]	0.07 (0.889) [-0.96,1.11]	1.16 (0.108) [-0.26,2.58]
**Intravesical BCG**									
**N**	64	67	131[e]	138[e]	62	53	115[e]	127[e]	
Logistic Regression on Log Odds Scale for Binary Outcome									
**GRA Responder[a]**	11 (17%)	14 (21%)	0.30 (0.494) [-0.55,1.15][f]	0.31 (0.471) [-0.54,1.16][f]	5 (8%)	13 (25%)	1.32 (0.020) [0.21,2.43][f]	1.18 (0.037) [0.07,2.28][f]	0.86 (0.225) [-0.53,2.26]
Absolute Change on NRS Scale									
**Change in Pain NRS[b]**	-0.96 (2.27)	-1.40 (2.61)	-0.39 (0.337) [-1.18,0.41]	-0.40 (0.311) [-1.18,0.38]	-0.86 (1.97)	-1.50 (2.21)	-0.63 (0.146) [-1.48,0.22]	-0.54 (0.195) [-1.36,0.28]	-0.14 (0.807) [-1.27,0.99]
**Change in Urgency NRS[b]**	-0.98 (2.03)	-1.57 (2.37)	-0.60 (0.111) [-1.34,0.14]	-0.60 (0.103) [-1.32,0.12]	-0.89 (1.96)	-1.48 (2.29)	-0.64 (0.114) [-1.43,0.15]	-0.52 (0.183) [-1.28,0.24]	0.08 (0.880) [-0.97,1.13]

	Low Widespreadness				High Widespreadness				
	Complete Cases			Intent to Treat	Complete Cases			Intent to Treat	
	**Observed Change: Control mean (SD)**	**Observed Change: Treatment mean (SD)**	**Complete Case Model: Treatment[c]**	**MI Model: Treatment[c]**	**Observed Change: Control mean (SD)**	**Observed Change: Treatment mean (SD)**	**Complete Case Model: Treatment[c]**	**MI Model: Treatment[c]**	**MI Model: Treatment Interaction[d]**
**Oral Amitriptyline with EBMP**									
**N**	75	74	149[e]	171[e]	44	37	81[e]	99[e]	
Logistic Regression on Log Odds Scale for Binary Outcome									
**GRA Responder[a]**	44 (59%)	49 (66%)	0.35 (0.309) [-0.33,1.03][f]	0.30 (0.379) [-0.36,0.96][f]	17 (39%)	25 (68%)	1.13 (0.017) [0.20,2.06][f]	1.18 (0.009) [0.29,2.07][f]	0.88 (0.118) [-0.22,1.99]
Absolute Change on NRS Scale									
**Change in Pain NRS[b]**	-2.64 (2.37)	-2.70 (2.43)	-0.04 (0.910) [-0.76,0.68]	0.05 (0.892) [-0.66,0.75]	-1.59 (2.38)	-2.45 (2.62)	-1.08 (0.032) [-2.06,-0.09]	-1.14 (0.019) [-2.08,-0.19]	-1.19 (0.049) [-2.37,0.00]
**Change in Urgency NRS[b]**	-2.81 (2.44)	-3.10 (2.49)	-0.21 (0.567) [-0.93,0.51]	-0.07 (0.835) [-0.77,0.63]	-1.99 (2.72)	-2.96 (2.53)	-0.79 (0.110) [-1.76,0.18]	-0.80 (0.103) [-1.75,0.16]	-0.72 (0.230) [-1.90,0.46]
**Change in Frequency NRS[b]**	-3.06 (2.35)	-3.52 (2.21)	-0.39 (0.298) [-1.12,0.34]	-0.24 (0.514) [-0.96,0.48]	-1.85 (2.61)	-3.50 (2.44)	-1.48 (0.004) [-2.47,-0.49]	-1.53 (0.002) [-2.50,-0.56]	-1.29 (0.036) [-2.49,-0.08]

[a] No. (%)

[b] Change is calculated as the outcome at the end of study subtracted from averaged baseline

[c] Estimated treatment effect (Change in outcome on treatment compared to control within widespreadness subgroup) from model results: Effect (p-value) [95% CI]

[d] Test for interaction between treatment effect and widespreadness category. Based off of multiple imputation model results. Difference=treatment high widespreadness-treatment low widespreadness (p-value) [95% CI]

[e] Sample size of treatment and control within widespreadness subgroup

[f] Treatment effect log odds ratio from model results: Log odds ratio (p-value) [95% CI]

Supplementary Table 3. Estimated change in NRS measure at end of study from average baseline for the average RCT participant

Estimated Change in Outcome from Baseline for Average RCT Participant							
		**Low Pain Widespreadness**		**High Pain Widespreadness**		**Combined**	
		**Placebo**	**Treatment**	**Placebo**	**Treatment**	**Placebo**	**Treatment**
	**Average Baseline Outcome**	**Estimated Change and 95% CI**					
**Oral PPS and Hydroxyzine**							
**Change in Pain NRS[a]**	6.05	-0.59 [-1.11,-0.08]	-2.04 [-3.11,-0.97]	-0.79 [-1.46,-0.11]	-0.95 [-1.96,0.06]	-0.65 [-1.05,-0.25]	-1.48 [-2.20,-0.75]
**Change in Urgency NRS[a]**	6.55	-0.96 [-1.40,-0.52]	-2.04 [-2.92,-1.17]	-0.82 [-1.39,-0.26]	-0.75 [-1.62,0.13]	-0.89 [-1.23,-0.55]	-1.43 [-2.04,-0.82]
**Intravesical BCG**							
**Change in Pain NRS[b]**	6.78	-1.03 [-1.59,-0.46]	-1.43 [-1.98,-0.88]	-0.77 [-1.34,-0.20]	-1.31 [-1.91,-0.71]	-0.89 [-1.28,-0.49]	-1.38 [-1.78,-0.98]
**Change in Urgency NRS[b]**	7.01	-1.04 [-1.56,-0.51]	-1.64 [-2.15,-1.12]	-0.79 [-1.31,-0.26]	-1.31 [-1.87,-0.74]	-0.92 [-1.28,-0.55]	-1.48 [-1.86,-1.11]
**Oral Amitriptyline with EBMP**							
**Change in Pain NRS[c]**	5.87	-2.89 [-3.40,-2.37]	-2.84 [-3.34,-2.34]	-1.02 [-1.68,-0.36]	-2.16 [-2.87,-1.44]	-2.17 [-2.57,-1.76]	-2.62 [-3.04,-2.21]
**Change in Urgency NRS[c]**	6.39	-3.14 [-3.66,-2.63]	-3.22 [-3.71,-2.73]	-1.64 [-2.29,-1.00]	-2.44 [-3.17,-1.71]	-2.52 [-2.92,-2.12]	-2.98 [-3.40,-2.57]
**Change in Frequency NRS[c]**	6.79	-3.30 [-3.83,-2.77]	-3.54 [-4.06,-3.02]	-1.60 [-2.26,-0.94]	-3.13 [-3.86, -2.40]	-2.63 [-3.04,-2.21]	-3.41 [-3.84,-2.97]
[a] Evaluated at average baseline outcome and for average Oral PPS and Hydroxyzine Trial participant (White=.843,Age=45.9,Female=.893)							
[b] Evaluated at average baseline outcome and for average Intravesical BCG Trial participant (White=.868,Age=47.65,Female=.819)							
[c] Evaluated at average baseline outcome and for average Oral Amitriptyline with EBMP Trial participant (White=.716,Age=39.01,Female=.833)							

Supplementary References

1. Wilkerson MD, Hayes DN. ConsensusClusterPlus: a class discovery tool with confidence assessments and item tracking. *Bioinformatics*. Jun 15 2010;26(12):1572-3. doi:10.1093/bioinformatics/btq170
2. Monti S, Tamayo P, Mesirov J, Golub T. Consensus Clustering: A Resampling-Based Method for Class Discovery and Visualization of Gene Expression Microarray Data. *Machine Learning*. 2003/07/01 2003;52(1):91-118. doi:10.1023/A:1023949509487
3. O'Leary MP, Sant GR, Fowler Jr FJ, Whitmore KE, Spolarich-Kroll J. The interstitial cystitis symptom index and problem index. *Urology*. 1997;49(5):58-63.
4. Goin JE, Olaleye D, Peters KM, Steinert B, Habicht K, Wynant G. Psychometric analysis of the university of Wisconsin interstitial cystitis scale: Implications for use in randomized clinical trials. *J Urol*. Mar 1998;159(3):1085-1090. doi:Doi 10.1016/S0022-5347(01)63840-0
5. Senbabaoglu Y, Michailidis G, Li JZ. Critical limitations of consensus clustering in class discovery. *Sci Rep-Uk*. Aug 27 2014;4:6207. doi:10.1038/srep06207
6. Warnes GR, Bolker B, Bonebakker L, et al. gplots: Various R programming tools for plotting data. *R package version*. 2009;2(4):1.
7. Gallop RJ, Crits-Christoph P, Muenz LR, Tu XM. Determination and Interpretation of the Optimal Operating Point for ROC Curves Derived Through Generalized Linear Models. *Understanding Statistics*. 2003/11/01 2003;2(4):219-242. doi:10.1207/S15328031US0204_01
8. Youden WJ. Index for rating diagnostic tests. *Cancer*. 1950;3(1):32-35.
9. Schenker N, Taylor JMG. Partially parametric techniques for multiple imputation. *Comput Stat Data An*. Aug 10 1996;22(4):425-446. doi:Doi 10.1016/0167-9473(95)00057-7
10. Van Buuren S. *Flexible Imputation of Missing Data.* Chapman & Hall/CRC.; 2012.
11. Rubin DB. *Multiple Imputation for Nonresponse in Surveys.* John Wiley & Sons; 1987.
12. Wickham H. *ggplot2: Elegant Graphics for Data Analysis.* Springer-Verlag; 2016.
13. Laupacis A, Sackett DL, Roberts RS. An assessment of clinically useful measures of the consequences of treatment. *New England journal of medicine*. 1988;318(26):1728-1733.
14. Hollander M, Wolfe DA, Chicken E. *Nonparametric statistical methods*. John Wiley & Sons; 2013.
